# Dendritic Cells: A Spot on Sialic Acid

**DOI:** 10.3389/fimmu.2013.00491

**Published:** 2013-12-27

**Authors:** Hélio J. Crespo, Joseph T. Y. Lau, Paula A. Videira

**Affiliations:** ^1^CEDOC – UC Imunologia, Faculdade de Ciências Médicas, Universidade Nova de Lisboa, Lisbon, Portugal; ^2^Department of Molecular and Cellular Biology, Roswell Park Cancer Institute, Buffalo, NY, USA

**Keywords:** dendritic cell, sialic acid, sialylation, lectins, host-pathogen interaction

## Abstract

Glycans decorating cell surface and secreted proteins and lipids occupy the juncture where critical host–host and host-pathogen interactions occur. The role of glycan epitopes in cell–cell and cell-pathogen adhesive events is already well-established, and cell surface glycan structures change rapidly in response to stimulus and inflammatory cues. Despite the wide acceptance that glycans are centrally implicated in immunity, exactly how glycans and their changes contribute to the overall immune response remains poorly defined. Sialic acids are unique sugars that usually occupy the terminal position of the glycan chains and may be modified by external factors, such as pathogens, or upon specific physiological cellular events. At cell surface, sialic acid-modified structures form the key fundamental determinants for a number of receptors with known involvement in cellular adhesiveness and cell trafficking, such as the Selectins and the Siglec families of carbohydrate recognizing receptors. Dendritic cells (DCs) preside over the transition from innate to the adaptive immune repertoires, and no other cell has such relevant role in antigen screening, uptake, and its presentation to lymphocytes, ultimately triggering the adaptive immune response. Interestingly, sialic acid-modified structures are involved in all DC functions, such as antigen uptake, DC migration, and capacity to prime T cell responses. Sialic acid content changes along DC differentiation and activation and, while, not yet fully understood, these changes have important implications in DC functions. This review focuses on the developmental regulation of DC surface sialic acids and how manipulation of DC surface sialic acids can affect immune-critical DC functions by altering antigen endocytosis, pathogen and tumor cell recognition, cell recruitment, and capacity for T cell priming. The existing evidence points to a potential of DC surface sialylation as a therapeutic target to improve and diversify DC-based therapies.

## Introduction

Immunological studies, nowadays, imply researchers have at least basic knowledge of glycobiology since, at some point of their study, researchers are faced with glycosylation-related features. Glycosylation is a post-translational modification of basically all the secreted and cell surface proteins, as well as of lipids. Thus, all contacts between cell surface and/or serum molecules are continuously accompanied by glycosylation. The immune response lays on innumerous contacts between cells and molecules, a good example being the case of immunological synapses, a junction that forms between T cells and specialized cells and the antigen-antibody interactions. All the immune encounters have, with great probability, glycans occupying, and influencing the juncture. Thus, all self-asserted immunologist should consider to be (at least partially) glycobiologists.

**Figure 1:**
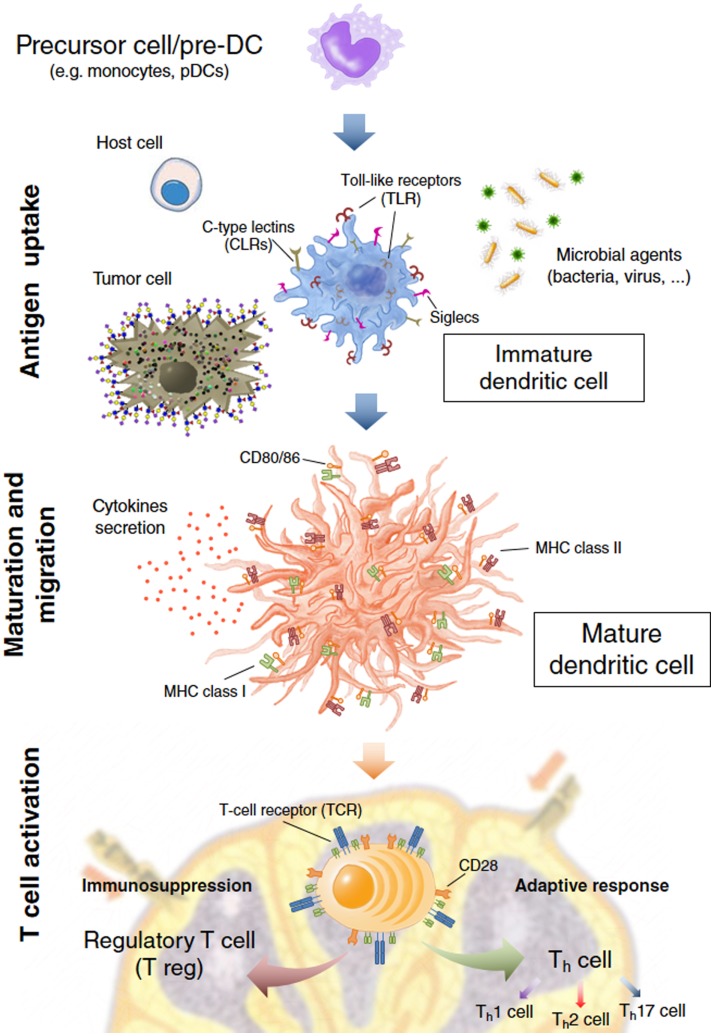
**Dendritic cell (DC) immune functions**. DCs act on three main events: the *antigen capture* after interaction with host cells, microbial agents, and tumor cells by recognizing Pathogen-Associated Molecular Patterns (PAMPs) and self molecules through Pathogen Recognition Receptors (PRRs) and other cell surface receptors like Siglecs or C-type Lectins (CLRs); *maturation and migration* toward the secondary lymphoid organs; *T cell activation* where DCs present the processed antigens to T cells eliciting a specific and enduring response or tolerance from T cells.

Among the several cell types that constitute the immune system, dendritic cells (DCs) are key players. DCs survey the microenvironment where they are positioned in order to help correctly classify collected antigen information, in a “self” or “‘foreign” category, and to respond accordingly. They carry antigen information from the infection site to the secondary lymphatic organs, presenting them to T cells, strongly potentiating a specific immune response against pathogens (Figure 1). The immune response is thus tremendously dependent on DCs and impairment of DC functions, as studied using animal models deficient for DC function-related molecules, or absence of DC populations, have been associated with infection or, oppositely, to a wide range of autoimmune diseases (1). DCs also play an important role in anti-tumoral immunity, whereupon specific cytotoxic T cells may be primed by DCs to respond against tumor cells. Investigating the underlying mechanisms of DC-pathogen or DC-host and -tumor cell interactions may help us to better comprehend the immune response in physiological and pathological events and to identify new targets for therapeutic intervention.

Dendritic cells show specific glycan patterns at cell surface, which are modulated during cell differentiation and respond to stimuli such as inflammatory cytokines and pathogens (2–4). Sialic acid is a sugar that frequently terminates glycan structures. Due to its terminal position and properties, sialic acid can mediate many immune processes such as host-pathogen recognition, migration, and antigen presentation, among other non-immune related processes. The addition of this sugar is mediated by a number of enzymes, the sialyltransferases, mainly located in the Golgi apparatus. Sialyltransferase expression is finely regulated during DC differentiation and maturation, concurring with the expression of sialylated structures (3, 4). In diverse immune events, the sialylated glycans will be recognized by lectins, i.e., carbohydrate-binding proteins that are expressed in other cells or by DCs. While promoting cell recognition by some lectins, the presence of specific sialic acids can actually switch off recognition by other lectins specific for asialylated glycans. Thus, glycan recognition by DC lectins may impact the DC immunobiological functions. Thus, a deeper understanding of sialic acid’s influence in the DC immunobiology potentially leads to a better understanding of the immune mechanisms mediated by DCs.

This review will focus on DC’s glycoimmune processes, with special attention to the sialic acid-mediated ones and how they modulate the different DC functions. It includes an introduction of DCs’ function and glycan recognition receptors, following a description of processes known to be mediated by sialic acid such as endocytosis, migration, priming of adaptive immune response, and pathogen/tumoral recognition.

## Dendritic Cells

Dendritic cells are part of the innate response and are essential to boost and/or regulate the adaptive immune response. They capture antigens in an earlier phase, process them “on the go” while migrating toward secondary lymphoid organs, such as lymph nodes, where they present, via major histocompatibility complex (MHC), the processed antigens to T cells and thus enacting an adaptive immune response. DCs can also present antigens to B cells, although by non-classical (non-MHC) mechanisms (5–7). Phenotypically, DCs are a heterogeneous population with different cell subsets, populating various organs. They can be broadly classified according the inflammatory status and differentiation state. Accordingly, conventional DCs are seen in a steady-state, that is, in the absence of infection and inflammation, and they can functionally be divided in two major types: migratory and non-migratory (lymphoid-tissue-resident) DCs [reviewed by Shortman and Naik (8)]. A good example of the former are dermal DCs and Langerhans cells that mainly reside in skin tissues and after antigen contact, they mature and migrate to the draining lymph nodes – hence the “migratory” classification. Conventional, non-migratory DCs (like spleen DCs) reside in secondary lymphoid organs, where they constantly screen blood or lymph for pathogens. The variety of DCs inside both these groups is significant and adapted toward the tissue where they reside in the immature state. Regarding DC differentiation, both canonical myeloid and lymphoid hematopoietic progenitors contribute to the steady-state DC pool and, actually, DCs use unique and flexible developmental programs that cannot be categorized into the conventional myeloid or lymphoid pathway. The expression of the Fms-like tyrosine kinase 3 (Flt-3) molecules is characteristic of DC precursors, regardless of the myeloid or lymphoid lineage and DCs development is driven by Flt3-ligand (Flt3L) (8–14). Much interestingly, it was recently reported that conventional DCs are marked by the exclusive expression of the DNGR-1 (15).

Opposed to the conventional DCs, some populations are inflammatory or infection-derived DCs. These populations include the plasmacytoid DC (pDC) population, a first line of defense against microbial invasion. Functionally specialized in the detection of viral infections, pDCs, develop a fully differentiated DC phenotype after infection and secretion of type 1 interferon (16, 17). Other inflammatory DCs include the monocyte-derived DCs (moDCs), comprising the TNF-α, inducible nitrous oxide synthase-producing DCs (Tip-DCs), a pathogenic subpopulation generated in an infection context (non-steady-state) [reviewed in Ref. (18)].

Dendritic cells constitutively uptake antigens in its surroundings as a surveillance measure (typical of the steady-state), fundamental to rapidly trigger the adaptive response against pathogens (inflammation) (19). DCs are, thus, naturally equipped with distinct means to uptake antigens, including: (1) receptor-mediated endocytosis, on which particles are endocytosed after cell surface receptor recognition; (2) macropinocytosis, or the non-selective endocytosis of solutes, a process constitutive in DCs and the major source of antigens for DC presentation (20); and (3) phagocytosis, the uptake of large molecules or cells, including virus, bacteria, protein clusters, apoptotic, and necrotic cells, which also involves specific membrane receptors. The uptake of foreign antigens usually trigger activation signals that will lead DCs to a mature phenotype, on which all the potential for antigen presentation and stimulation of the adaptive response immune cells is maximized.

Endocytosis is also fundamental in the maintenance of the self-tolerance mechanisms since, at steady-state, self-antigens are normally endocytosed and posteriorly presented by DCs. Endocytosis of self-antigens does not usually induces significant maturation changes (21), thus contributing to turn DCs tolerogenic and promoting regulatory but not effector T cells. Nevertheless, it has been suggested that the presence of very small, time-persistent concentrations of foreign and more common antigens are responsible for the induction of tolerance to those same antigens. These tolerance-inducing antigens are expressed by microorganisms present during the development of the immune system, such as commensal bacteria, flora members, and helminthes. The knowledge about these mechanisms raised the hypothesis that common microorganisms are able to regulate the immune system, the “old friends” hypothesis (22–24). These time-persistent antigens, thus shape our immune system to its present state, being presently not only tolerated but, in fact, needed in order to maintain the general tolerance balance. The “old friend” hypothesis complements the “hygiene” hypothesis stating, in brief, that the lack of immune challengers due to excessive hygiene is related to the growing number of autoimmune and hypersensitivity diseases that is observed in the developed countries, and not in the developing ones (24). Due to its key role in antigen uptake and presentation, DCs too may be involved in this mechanism of tolerance-induction toward these “old friends.”

Dendritic cell maturation is the sum of all the phenotypical and functional changes occurring upon encounter with immune stimuli (i.e., antigens, cytokines, etc.) and it is crucial to enable DCs to effectively activate T cells. It is characterized by rapid downregulation of the antigen uptake process, acidification of lysossomal compartments, higher expression of MHC II molecules and of CD80 and CD86 co-stimulatory molecules, *de novo* or upregulated synthesis of DC-specific inflammatory cytokines (25). All these maturation and migration-changes are necessary hallmarks to enable DCs to perform antigen presentation and boosting T and B cell responses (26). It is also known that the molecular nature of uptaken antigens, as well as the cytokines to which DCs are exposed during the uptake process, are responsible for the modulation of the maturation process. This ultimately influences the differentiation of the DC-pulsed T cells into functionally distinct subtypes, namely, T helper type 1 or 2 (Th1 or Th2), T helper 17 (Th17), or regulatory (T_reg_) cells, actively shaping a future active or tolerance response.

The migration (or homing) of conventional or inflammatory DCs loaded with antigens to T cell niches (normally, secondary lymphatic organs) is a crucial step for the setting of effective immune responses. This process is characterized by chemokine-mediated cell recruitment to the lymphoid target site and, activation of the surrounding tissues (27–29). Tissue activation helps to increase the cell adhesion to the endothelium, by inducing the expression of several adhesion molecules, of which integrins and selectins and its ligands are the most relevant elements.

From all the above observations, it is, thus, clear and generally accepted that DC functions rely on a complex set of mechanisms that involve DC differentiation, ontogeny, maturation, and permanent contacts with other cells and pathogens.

### Pathogen recognition by dendritic cells

Pathogen recognition by DCs depends on the identification of distinct microbial patterns, not present in mammalian cells, but shared by most of the pathogenic microbial, known as “pathogen-associated molecular patterns” (PAMPs) (30, 31). They include bacterial and viral unmethylated CpG DNA, bacterial flagellin, peptides containing *N*-formylmethionine residues, lipoteichoic acids, and double-stranded and single-stranded viral RNA. A substantial part of PAMPs are glycan-containing ones, such as lipopolysaccharide (LPS), *N*-acetylglucosamine, peptidoglycan, and terminal fructose- and mannose-containing glycans, and glucan-containing cell walls from fungi.

Pathogen-associated molecular patterns are recognized by specific receptors named “pattern recognition receptors” (PRRs), with functions aggregating endocytosis and intracellular signaling. Examples of PRRs expressed by DCs include Scavenger receptors, Nod-like receptors, and C-type lectins (CLRs). However, perhaps, the most widely studied are the Toll-like receptors (TLRs), a growing family of 12 evolutionary conserved PRRs consisting of type 1 integral membrane glycoprotein with relevant role in the microbial response. The outcome of TLR recognition is the induction of intracellular signaling and consequent expression of antigen presentation molecules (MHC II molecules), co-stimulatory molecules (CD80/86, CD40), inflammatory and/or antiviral cytokines (such as TNF-α, IL-12, IL-23, IFNα/β), chemokines (i.e., IL-8, RANTES) (32, 33), thus enacting a powerful response against pathogenic microbes.

C-type lectins are another very relevant family of PRRs expressed by DCs (34). Being lectins, their main function is to recognize glycan structures and, in immunological context, they recognize pathogen-associated glycans or glycosylated self-antigens. In DCs, some CLRs of note include the DC-Specific Intracellular adhesion molecule-3 Grabbing Non-integrin (DC-SIGN), CD207/Langerin, the Selectin family (discussed below), the Macrophage Galactose/*N*-acetylgalactosamine-specific Lectin (MGL-1), Mannose Receptor (MR), DEC205, the Blood DC antigens 2 (BDCA 2), the Dendritic Cell Immunoreceptor (DCIR), the Dendritic Cell Immunoactivating receptor (DCAR), and Dectin-1/2/3. In contrast to TLRs, all of these CLRs functionally bind glycan structures expressed by mammalian cells (except for Dectin-1/2/3 that apparently only recognizes fungal and/or mycobacterial glycans), a fact demonstrating its potential role in both host and pathogen recognition (35). CLRs can also recognize and internalize pathogens for presentation without inducing DCs’ maturation. In fact, the CLR-mediated antigen uptake doesn’t necessarily elicit a factual immune response, and may instead contribute to induce immunological tolerance (36). A downside of these phenomena is the potential immune escape of pathogens recognized via CLRs (35, 37–39).

Like CLRs, the Sialic acid-binding immunoglobulin-like lectins (Siglecs) can also recognize pathogens’ glycoproteins and glycolipids thus also contributing to the host’s innate immune responses. Siglecs specifically recognize sialic acid-containing glycans and as mentioned below they also play a relevant role in self recognition (40–43). The biological and immunological relevance of CLR and Siglec receptors will be discussed in detail in later sections.

Dendritic cells can also recognize and internalize microbes and its derivate particles by receptors that bind to opsonins in opsonized (“coated”) microbes. Opsonization of microbes can occur in two forms: by coating with complement proteins or by binding of antibodies to antigens expressed on their surface. DC recognition of opsonized microbes is thus mainly mediated by complement receptors and Fc receptors and assures the capture of pathogens that might otherwise evade recognition by other DC receptors (44, 45).

Summarizing, DCs can interact in different ways with microbes, as well as with the host antigens, through panoply of receptors. This recognition initiate mechanisms that will induce or suppress a specific immune response. The DC recognition is thus considered to be of great relevance for the development of a suitable, specific immune outcome, dictating the balance tolerance/reactivity of the developing host-pathogen response.

### Dendritic cells-based therapy

The current knowledge of DC immunobiology allowed several biotechnological and pharmaceutical companies to develop DC-based immunotherapies. Applications for DC-based therapy include a plethora of pathologies ranging from infectious and hypersensitivity diseases to malignancies. One strategy is the *ex vivo* upload of DCs with the antigen to turn them able to efficiently develop an efficient response against the antigen bearer (46–50). The best example of this strategy is the vaccination of cancer patients with DCs loaded with tumor antigens.

Other approaches include the use of specific antibodies targeting DC endocytic receptors that are used to force the upload of specific antigens toward that receptor. Antibodies are also used to block specific receptor-ligand interaction and consequent downstream signaling, counteracting for instance the negative immunomodulatory cues of the tumor microenvironment.

Dendritic cells have also been studied as targets of DNA vaccines encoding for antigens (51). Viral transduction not only targets antigens to DCs, but also induces intracellular pathways to modulate the immune response (52).

All these relatively recent drug-niche that exploits DC unique immune potential is proof of reconnaissance of DCs’ cornerstone role in the immune system. Nevertheless, the DC-based therapies still face several hindrances to full application, mostly derived from the lack of full knowledge regarding pathogenesis/tolerance balance mechanisms, an area where glycosylation has been shown to have a relevant role.

## Glycosylation and Sialylation

Glycosylation is the most frequent modification of proteins and lipids. The majority of glycans exist as membrane-bound or soluble glycoconjugates. One consequence of this fact is that all cells present at their surface a glycocalyx, that is, the full surface-complex of glycans, glycoproteins, and glycosylated lipids. The three main classes of glycoconjugates are glycoproteins, proteoglycans, and glycolipids and their synthesis occurs mainly in (but not limited to) the lumen of the endoplasmic reticulum and in the Golgi apparatus. In glycoproteins, the sugar chain is classified as *N*- or *O*-linked, depending if the glycosidic moiety is linked to an asparagine (Asn) residue in the protein moiety or to a serine/threonine (Ser/Thr) residue, respectively.

The cell glycocalyx is the result of many factors. The most relevant one is probably the expression of the set of enzymes responsible for the synthesis and/or transfer of glycosylated structures, i.e., the glycosyltransferases. Also critical is the expression of enzymes responsible for the removal of glycans or entire structures from glycosylated molecules, i.e., the glycosidases. These two sets of enzymes work in a finely controlled balance both during the glycoconjugate synthesis at the Golgi apparatus. Both enzyme types can also be present in plasma membrane or soluble forms, with potentially relevant biological roles as we shall see in sections below (53–56).

Sialic acids are a large family of negatively charged, nine-carbon monosaccharides that are normally found at glycan terminal positions. They include *N*-acetylneuraminic acid (Neu5Ac), *N*-glycolylneuraminic acid (Neu5Gc), and 9-*O*-acetyl-*N*-acetylneuraminic acid (9-*O*-Ac-NeuAc). Human cells can only synthesize Neu5Ac. However, Neu5Gc can also be found in some tumor cells (57). Interestingly, some pathogens may express Neu5Ac, but Neu5Gc has never been reported to be synthesized by any pathogenic bacteria (58). This review will focus mainly on Neu5Ac and, for the sake of simplification, and we will strictly refer to Neu5Ac when using the term “sialic acids.”

Sialyltransferases are a family of twenty glycosyltransferases that catalyze the addition of sialic acids to terminal non-reducing position of the oligosaccharide, transferring the sialic acid from the activated sugar donor CMP-Neu5Ac to different sugar acceptors (Table 1). Sialyltransferases normally locate at the Golgi apparatus as integral membrane proteins adding sialic acids to glycoconjugates during their synthesis. However, some sialyltransferases are also expressed as soluble enzymes (59) and sialyltransferase activity at plasma membrane has been reported in immune cells (54). Each sialyltransferase presents high selectivity toward its acceptor substrate. *In vivo*, competition between other sialyltransferases and glycosyltransferases’ common substrates is observed and, as a result, the cell’s sialylation status is the dynamic sum of transferase activities, Golgi localization, and concentration of activated sugar donors. Sialyltransferases depending on their specificities, can establish α2,3-, α2,6-, α2,8-linkages and can be organized in four families depending on linkage specificity and acceptor substrate: the ST3Gal family, catalyzing the addition of sialic acid to a terminal galactose of *O*-linked glycans and glycolipids in an α2,3-linkage; the ST6Gal family, α2,6-linking sialic acids to galactose residues of *N*-glycans; the ST8Sia family, the only known sialyltransferases promoting the linkage to another sialic acid residue in *N*- or *O*-glycans, in a α2,8-bond; and, finally, the ST6GalNAc family, adding sialic acid to terminal *N*-acetylgalactosamine (GalNAc) residues of glycoproteins and glycolipids, in an α2,6-linkage (60). Thus, on the cell surface, sialic acid residues can be present in *N*- and *O*-glycans in glycoproteins, as well as in gangliosides, i.e., a glycolipid containing one or more residues of sialic acid.

**Table 1:** **Human sialyltransferases and sialidases**.

	Preferred saccharide substrate	Glycan specificity	Dendritic cell expression (cell status)
**SIALYLTRANSFERASE**			
ST3Gal-I	Galβ1,3GalNAc	*O*-glycan	Yes
ST3Gal-II	Galβ1,3GalNAc	*O*-glycan	Yes (mature)
ST3Gal-III	Galβ1,3(4)GlcNAc	*O*-glycan, *N*-glycan	Yes (mature)
ST3Gal-IV	Galβ1,4(3)GlcNAc	*N*-glycan, *O*-glycan	Yes (mature)
ST3Gal-V	Galβ1,4Glc-ceramide	Glycolipid	Yes
ST3Gal-VI	Galβ1,4GlcNAc	*N*-glycan	Yes
ST6Gal-I	Galβ1,4GlcNAc	*N*-glycan	Yes
ST6Gal-II	Galβ1,4GlcNAc	*N*-glycan	No
ST6GalNAc-I	GalNAcα1,*O*-Ser/Thr	*O*-glycan	No
	Galβ1,3GalNAcα1, *O*-Ser/Thr	
ST6GalNAc-II	Galβ1,3GalNAcα1, *O*-Ser/Thr	*O*-glycans	Yes
ST6GalNAc-III	Siaα2,3Galβ1,3GalNAc	*O*-glycan	Yes (?.)
ST6GalNAc-IV	Siaα2,3Galβ1,3GalNAc	*O*-glycan	Yes
ST6GalNAc-V	GM1b	Glycolipid	No
ST6GalNAc-VI	All α-series gangliosides	Glycolipid	Yes
ST8Sia-I	Siaα2,3Galβ1,4Glc-ceramide	Glycolipid	No
ST8Sia-II	Siaα2,3Galβ1,4GlcNAc	*N*-glycan on NCAM	No
ST8Sia-III	Siaα2,3Galβ1,4GlcNAc	*N*-glycan on NCAM	No
ST8Sia-IV	(Sialα2,8)_n_Siaα2,3Galβ1-R	*N*-glycan on NCAM	Yes
ST8Sia-V	GM1b, GT1b, GD1a, GD3	Glycolipid	No
ST8Sia-VI	Siaα2,3(6)Gal	Sialic acid on *O*-glycan	Unknown
**SIALIDASES**			
Neu1	Siaα2,3	Oligosaccharides	Yes
	Siaα2,6	Glycopeptides	
Neu2	Siaα2,3	Oligosaccharides	No
	Siaα2,6	Glycopeptides	
		Gangliosides	
Neu3	Siaα2,3	Gangliosides	Yes (mature)
	Siaα2,6	
Neu4	Siaα2,3	Oligosaccharides	Yes
	Siaα2,6	Glycopeptides including mucins	
		Gangliosides	

*Preferred substrates for each enzyme and expression pattern in human dendritic cells are indicated. Data was based on (2, 4, 61, 62)*.

*? stands for “unknown” regarding the cell status (whether mature or immature)*.

The overall sialic acid content of a cell is also regulated by the removal of sialic acid residues, catalyzed by the sialidase enzymes. Four known enzymes fit into this family, also known as the Neuraminidase family: Neu1, Neu2, Neu3, and Neu4. These sialidases are variedly distributed, with Neu1 located at the lysosomes and also expressed on the surface of diverse types of cells, Neu2 at the cytosol, Neu3 integrated in the cell membrane, and Neu4 being an intracellular protein. They are all exoglycosidases, i.e., they cleave terminal sialic acids, but have different substrate specificities: Neu1, Neu2, and Neu4 remove sialic acid residues from glycoproteins, Neu2 and Neu4 also cleaves sialic acids from glycolipids, and Neu3 preferentially hydrolyzes gangliosides. A list of human sialyltransferases and sialidases, their expression patterns in DCs, and their preferred acceptor and donor substrates, is shown in Table 1.

## Sialylation and Modulation of the Immune Response

The terminal position occupied by sialic acids on membrane and extracellular glycans puts them on the frontline during leukocyte communication and overall immune response. Sialic acids, on an immune perspective, can function in two (seemingly contradictory) major ways: as biological masks and as recognizable cell patterns (63). In the former way, sialic acid helps shield host cells from pathogen recognition. It also prevents autoimmune responses, by preventing complement deposition over cell surface. Furthermore, it was reported that, during acute phase inflammation, both soluble and cell surface sialic acid is increased, as a consequence of the increase in soluble and circulatory forms of sialyltransferases. Higher sialic acid is thus part of the acute phase response and it seems to protect cells against pathogens, and also helping the immune system distinguishing “self” from “non-self” antigens (64). ST6Gal-I is an example of sialyltransferase whose soluble expression is upregulated during inflammation and its expression has been used by some authors as a serological clinical marker for inflammation (65–67). Non-sialylated glycans are recognized by specific lectins, and the addition of sialic acid to its terminal position may blocklectin binding. As an example, the presence of α2,6-linked sialic acids on *N*-glycans blocks recognition by galectins (68), a family of β-galactoside-binding lectins that regulate diverse cell behaviors, such as cell adhesion, migration, proliferation, differentiation, transformation, apoptosis, angiogenesis, and immune responses (69–73). However, sialic acid masking can also be used by pathogens, as a mimicry tactic in order to evade the immune system. This is the case of some *Trypanosoma* spp. that have mutated ST3Gal sialyltransferases that act as trans-sialidases, transferring the host’s sialic acid to coat themselves in order to evade host recognition (74, 75).

Opposed to the asialylated-glycan recognition, sialic acids can be recognized by several cell surface receptors, such as the previously mentioned CLRs and Siglecs (41, 76). Siglecs are sialic acid-recognizing proteins that, albeit structurally similar, are commonly organized in two categories: (i) one comprises the CD22 family [including CD22 (or Siglec-2), sialoadhesin (or Siglec-1), myelin-associated glycoprotein (MAG or Siglec-4), and Siglec-15]; and (ii) the CD33-related family comprising CD33 (or Siglec 3), Siglec-5, -11, -14, and -16 in humans, all chiefly expressed in myeloid and lymphoid cells (63, 77). Siglecs recognize and bind ligands present not only in other cells (viz., in *trans*) but also on the same cell (in *cis*). Many Siglecs present one or two intracellular immunoreceptor tyrosine-based inhibitory motifs (ITIMs), classically described as being involved in signaling to regulation-inducing pathways, or intracellular tyrosine-based activation motifs (ITAMs), involved in the initiation of activation signaling pathways. Hence, Siglecs have a decisive role in regulating, positively or negatively, immune responses such as inflammation or tissue damage by actively discriminating between self-associated molecular patterns (SAMPs) and PAMPs (41, 63, 78, 79).

Studies using mice deficient for selected α2,3- and α2,6-sialyltransferases have provided evidence confirming the importance of sialic acid in immune processes (80–82). ST6Gal-I KO mice were reported as presenting impaired humoral immune response, namely, by reduced concentration levels of circulating and surface IgM, impaired B cell proliferation in response to various activation signals and impaired antibody production following contact with antigens (80). CD22, one of the first described Siglecs (83), was later shown to recognize ST6Gal-I-mediated glycans, functionally regulating several B cell functions and survival mechanisms (84). Other ST6Gal-I KO mice studies have also revealed that soluble forms of ST6Gal-I have a relevant role in myelopoiesis during acute inflammation, namely, by limiting it, thus avoiding uncontrolled excessive neutrophilic and eosinophilic inflammatory responses (59, 85, 86). Using ST3Gal-I KO mice, on the other hand, it has been shown that α2,3-sialylated *O*-glycans are required for CD8^+^ T cell homeostasis and survival (82).

These are only few examples on how sialic acids influence immune-relevant processes. Other examples include roles in host-pathogen interactions, regulation/modulation of the acute phase response and influence in the progression and differentiation of human malignancies.

## Dendritic Cells and Sialic Acid

As above mentioned, DCs play a role of enormous relevance in the immune system. Ever since Dr. Steinman and co-workers first described these cells (87–91), there has been an effort to fully characterize their immunobiology, and as part of those efforts, the relevance that glycosylation may have on it. The characterization of the DC’s “glycome” (“sialome” included) and its functional impact on the DCs immunobiology and, of course, on the immune system has been a work in progress. There are many questions still open, with many potential clinical applications.

### Sialylation in dendritic cells

In human DCs, the sialylation profile of inflammatory DCs has been the most studied. This comes as the result of two factors: first, they are the most frequent population of DCs and, second, in more practical terms, they are the easiest subset to obtain *in vitro* with human moDCs being a widely used human conventional migratory and inflammatory DC model. Other vertebrate DC models rely on the obtainment of DCs by differentiation of bone marrow extracts or, more specifically, CD34^+^ hematopoietic precursors myeloid lineage (92).

Immature moDCs present a high sialylation content, namely α2,3- and α2,6-sialylated glycoproteins, when compared to its monocyte precursors (4, 93). This has been reported by different teams that used plant lectins from *Sambucus nigra* and *Maackia amurensis*, preferably recognizing α2,6-linked sialic acid linked to lactosamine (Neu5Acα2,6Galβ1,4GlcNAc-) in *N*-glycans and α2,3-linked sialic acid linked to lactosamine (Neu5Acα2,6Galβ1,4GlcNAc-), respectively.

Quantitative Real-Time PCR and microarray analysis has shown that both sialyltransferases and sialidases undergo significant gene expression variation during differentiation and maturation (4, 62, 93, 94). In particular, a significant upregulation of the ST3Gal-I and ST6Gal-I genes occurs during moDCs’ differentiation that correlates with an increase of enzymatic activity by these two enzymes. Increased phenotypic change in α2,3- and α2,6-sialylation (4) during myeloid lineage-committed differentiation indicates these two sialyltransferases as the major contributors to the biosynthesis of α2,3- and α2,6-linked sialic acid-containing glycan structures specific for moDCs, with potential functional relevance. There are, however, other potentially relevant sialyltransferases that should not be discarded, such as ST3Gal-IV and -VI, being described as essential for the synthesis of the adhesion-related sialyl-Lewis x (sLe^x^) antigens. Regarding sialidases, modulation during moDCs’ differentiation is similarly observed, with Neu1 and Neu3 being significantly upregulated during this process (62). Maturation of moDCs leads to an increase of α2,3-sialylation and a decrease of α2,6-sialylation (2, 4, 93) although the reported variations are stimulus-dependent processes, and correlated with the sialyltransferases and sialidases activity.

While the functional impact of these observed sialic acid changes has to be further elucidated, there is already some evidence that these variations are biologically relevant, as it will be discussed further on in this review.

### Sialic acid-recognizing dendritic cell receptors

Sialic acid-containing glycans expressed by DCs are the target of receptors, such as Siglecs, being the largest represented family. Recognition of DC sialylated glycans has functional implications: examples include a recognition mechanism of high α2,6-sialic acid content of immature and tolerogenic DCs by inhibitory Siglecs expressed by effector T cells as a host-tolerance-inducing mechanism (93), or the observed increased binding of Siglecs-1, -2, and -7 correlating with the higher sialic acid content of mature DCs (2). All this gathered evidence point to an even more promising, relevant role of Siglec-mediated immunobiological processes involving DCs and other leukocytes, but still to be unraveled and requiring, thus, further studies.

Besides being recognized by Siglecs through their expression of glycans, DCs express themselves Siglecs enabling them to also recognize sialylated structures. MoDCs and blood-circulating DCs [namely pDCs, CD1a^+^, and CD141^+^ DCs (95)] express Siglec-1, -2, -3, -5, -7, -9, -10, -14, and -15 (2, 43, 78, 96), while pDCs have a more restricted pattern and apparently only express Siglec-5 (43). Siglecs, with the exception of Siglec-14 and -15, expressed by DCs present ITIM motifs in their cytosolic portion and are therefore mainly involved in inhibiting activation signals and have an immunoregulatory function (40, 41).

The concentration of sialic acids on surfaces of human cells is very high; for example, Stamatos and colleagues estimated that DCs had 8.9 nM per 5 × 10^6^ cells, which correspond to nearly 10^18^ sialic acid molecules per cell (62).

Therefore, it is possible that the majority of Siglecs expressed at DC surface bind *in cis*, i.e., to sialic acids at their own cell surface. The *cis* interaction will have primacy over the *trans* interactions, the only exception being sialoadhesin, which has an extended structure, projecting their binding site away from plasma membrane and being therefore involved in *trans* interactions (97).

Siglec interactions *in cis* can be released by sialidase activity, either extrinsic for instance from pathogens or intrinsically due to the activity of endogenous sialidases (40, 98). Since DCs ultimate function is to immunomodulate T cells and (to some extent) B cells, Siglecs potentially play a largely relevant role in host-tolerance mechanisms (2, 43, 99). Chen and co-workers reported Siglec-10 as involved in helping distinguish TLR-recognized danger-associated molecular patterns (DAMPs) – generated during cell/tissue damage or even regular cell lifecycle – from PAMPs, thus controlling inflammation (100). There are known examples of T cell activation where DC Siglecs have a relevant role in inducing Th1 and Th2 responses, as is the case of DC Siglecs-1 and -7 *trans* recognition of α2,3-sialic acids and α2,8-polisialic acids, respectively, in mimicked GM1a and GD1a (Siglec-1 recognized) and GD1c (Siglec-7 recognition) gangliosides included in *Campylobacter jejuni*’s LPSs (101).

CD33-related Siglecs can function as endocytic receptors that are important in the clearance of sialylated antigens. On the other hand, many pathogens are able to express appropriate sialic acids themselves (102–105). Pathogen’s sialic acids may interfere with DC functions such as endocytosis (43, 106) thus helping DCs to internalize and further present pathogen’s antigens. This, however, may also open an opportunity window for pathogens to modulate DCs’ immune functions (by binding to immunoregulating Siglecs) or even use DCs as vectors (i.e., “Trojan horses”) for infection of other immune system cells, such as HIV using Siglec-1 as a gateway-receptor for DC entry and posterior transmission to CD4^+^ T cells (107). However, a safety mechanism may be present: Siglec-15 can act as an activation receptor balancing the negative signaling triggered after recognition of sialylated pathogens (viz., enveloped viruses) through inhibitory Siglecs (78).

### Dendritic cell sialylation and endocytosis

The sialic acid’s role on endocytosis has long been studied on the perspective of the pathogen. Besides the already referred trans-sialidase bearing *T. cruzi* parasite, it is also known that several bacteria developed sialic acid-masking mechanisms in order to escape immune surveillance and/or response (108). Recent discoveries, however, hinted that sialic acid’s role in these immune processes goes far beyond than “just” being an antigen, with a functional impact on the innate immune phase cells as well, like DCs.

As previously mentioned DCs are functionally well prepared to endocytose pathogens, in order to process and present them to the adaptive immune response cells (20, 109, 110). Using two different approaches – sialidase treatment of moDCs and bone marrow-derived DCs (BMDCs) from sialyltransferase KO mice, it has been determined the functional impact of sialic acid on macropinocytosis and phagocytosis. Asialylated DCs presented significantly reduced ovalbumin-macropinocytosis but increased phagocytosis levels (111). Similar results having been obtained using BMDCs from ST6Gal-I and ST3Gal-I sialyltransferase-deficient mice (3).

Sialic acid removal (or absence in BMDCs) has a positive impact over the DC maturation process, leading to higher expression of maturation markers. Hence, this effect should account for the observed reduction of macropinocytosis levels, since matured DCs tend to have decreased endocytosis ability (112). The observed increase in phagocytosis in asialylated immature and mature DCs (111) seems, therefore, to oppose the endocytosis reduction induced by maturation. It is documented, however, that mature DCs may continuously uptake antigens by phagocytosis and receptor-mediated endocytosis, even if always described in lower levels than immature DCs (110, 113). As no studies have been performed from a sialic acid point of view, this can account for novel, groundbreaking evidence adding to the well-established concepts of endocytosis. Another piece of this apparent puzzle lies in the DC cytoskeleton, which has to be adjusted to perform cellular extensions needed for phagocytosis. After sialidase treatment of DCs, a cytoskeleton disorganization is observed. In addition, the activity of two Rho GTPases – Rac1 and Cdc42 – that regulate, among other processes, the actin-dependent events of macropinocytosis and phagocytosis (19, 112, 114–116) are downregulated, after sialidase treatment. This may justify the cytoskeleton disorganization and decreased macropinocytosis.

Hence, the fact that sialidase treatment accounts for the significant *E. coli* phagocytosis enhancement, in both sialidase-treated immature and mature DCs, is a process unrelated to maturation. Interestingly, the effect on phagocytosis seems to depend on the presence, in *trans*, of bacterial sialic acid (111), adding a potential involvement of Siglecs. Hence, sialidase treatment would release DCs’ Siglecs from in *cis* ligands, making them available to bind to ligands in *trans*, such as sialic acid-containing glycans present in the *E. coli* cell wall. However further investigations are still needed to elucidate the underlying mechanisms.

Sialidase-induced activation of receptors is not a novel phenomenon. Receptors like TLR-4 are known to depend on the activity of membrane sialidase prior to LPS-induced activation: in mice, DC phagocytosis is activated by desialylation of surface receptors (62, 117, 118). This evidence is in line with the already mentioned increased expression of sialidases, such as Neu1 during DCs differentiation and maturation (117). Furthermore, physiologically, cell surface sialic acid content is not exclusively cleaved by endogenous sialidases, since exogenous sialidase sources are also released by pathogenic bacteria or virus during the course of an infection. In the mouse model, it was reported that Neu1-induced desialylation activates phagocytosis by macrophages and DCs (118). Also, cell surface desialylation by influenza virus sialidase stimulates the internalization of target virus by infected mouse macrophages (119).

Siglecs and TLRs fit perfectly in the recent model presented by Cabral and collaborators showing that sialidase treatment of DCs favors phagocytosis (111). Since they are receptors with both strong activating- and suppressive-inducing properties, with known roles in regulating immune responses and with the potential of becoming active after sialic acid removal by sialidases, they are also likely to account for the observed upregulation of both pro- and anti-inflammatory cytokines (111). Nevertheless, the referred receptor families may not be the only receptors affected by sialidase treatment in DCs as novel glycan-protein interactions are continuously being revealed, but further studies are in order to better elucidate the role of sialic acid in endocytosis.

### Sialylation and dendritic cell migration

Dendritic cell migration includes both DC recruitment to non-lymphoid tissue and homing to lymphoid organs.

When located within tissues, DCs may respond to pro-inflammatory cytokines and pathogens, which trigger maturation and DCs then migrate to lymphoid tissues via afferent lymphatic vessels, wherein they activate antigen-responsive T cells. Immature and mature DCs may also enter the blood and from there disseminate to non-lymphoid and lymphoid organs, thereafter returning to blood, thus undergoing cycles of recirculation. Therefore DCs have complex trafficking routes, allowing for dynamic reassortment of DCs, making the most of their capacity to uptake antigens and to encounter T cells to present antigens and activate them.

While, generally, the migratory processes are based upon mechanisms like adhesion and chemotaxis, some processes still show their own particularities. The extravasation of blood DCs to any tissue involves DC adhesion to endothelium and is dependent of selectin interactions with sialofucosylated glycans. The role of certain sialylated glycans as selectin ligands is one of the most recognized functions of sialic acid in the context of leukocyte recruitment (120).

Selectins are CLRs expressed by platelets, endothelium, or leukocytes, hence taking their name: P-, E-, or L-selectins, although endothelial cells also express P-selectins.

All selectins recognize the sialic acid and fucose (Fuc) containing tetrasaccharide, where sLe^x^ (Neu5Acα2,3Galβ1,4[Fucα1,3] GlcNAc-) is the major prototype. Selectin ligands are expressed in most circulating immune cells and some endothelial cells during inflammation. They mediates essentially the rolling and tethering phase of cell transmigration over the endothelial cell surface (121). sLe^x^ expression is well characterized in neutrophils and lymphocytes (76, 122) but only relatively approached in DC (123–126). Recently, it was found that moDCs also express functional selectin ligands, based on their observations of moDC tethering and rolling over a P-, E-, and L-selectin immobilized surface (126). They observed decreasing tethering affinities (by decreasing order) toward P-, E-, and L-selectin, with similar lower rolling velocity on P- and E-selectins and the largest rolling velocity observed over L-selectins. These findings were in line with other studies using blood DCs and CD34^+^-derived DCs (125, 127). Furthermore, the use of anti-sLe^x^ antibodies on the rolling studies resulted in a significant binding inhibition, definitely proving that sLe^x^ mediates the moDCs-selectin binding (126).

**Figure 2:**
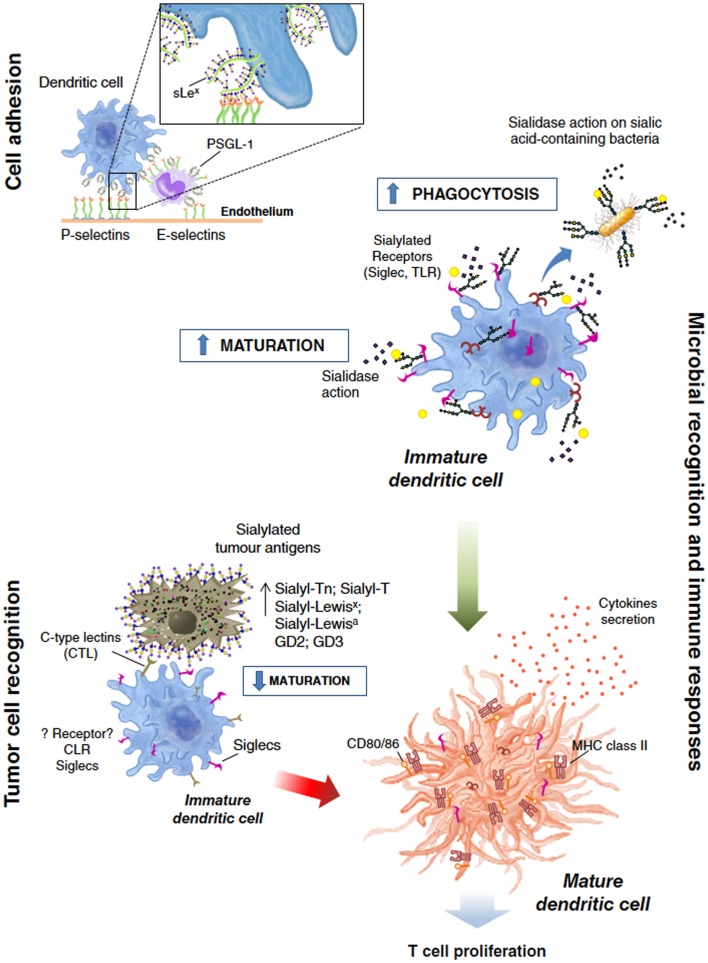
**General overview of the dendritic cell functions modulated by sialylation**. Sialic acid-containing glycans actively participate and modulate processes like: *cell adhesion* during migration and homing; or in “de facto” immune processes such as *tumor cell recognition* and *microbial recognition*, overall modulating the immune response/tolerance balance.

In order to properly function as a selectin ligand, sLe^x^ must be expressed in carrier glycoproteins or glycolipids (128, 129). The only described sLe^x^ carrier-protein described in moDCs is the P-selectin glycoprotein ligand-1 (PSGL-1) (123), a mucin-like glycoprotein, present in the microvilli of most leukocytes (130, 131) (Figure 2, “Cell adhesion” feature). In DCs, sLe^x^-decorating PSGL-1 is the solely ligand for P-selectin, with significant less affinity toward L-selectin and being indifferent for E-selectin binding (126).

Nevertheless, sialic acids also participate in the chemokine receptor-mediated firm arrest, as well as in β1 integrins function (132–134). There is also evidence concerning the chemokine-mediated migration to the lymph nodes. It was recently reported that ST8Sia-4-dependent polysialylation of neuropilin-2 seems to be relevant for chemokine-driven migration toward lymph nodes (135). Other report claims that ST3Gal-IV is not relevant for chemokine-dependent DC homing, in the mouse model (120), but, interestingly, our team’s preliminary studies using ST6Gal-I-deficient mice have shown impaired DC migration toward draining lymph nodes, suggesting a previously unknown role for α2,6-sialylated *N*-glycans in DC homing.

Dendritic cell mobility is a crucial step still needing to be better elucidated. Most of the clinically efficacy of DC immunotherapy relies on the migration ability of these cells. In *ex vivo* generated DC vaccines, it is estimated that only 1–2% of total administered DCs reach secondary lymphatic organs (136). Therefore the majority of *ex vivo* generated DCs are inefficient because they do not meet T cells. Thus, understanding DC migration should be regarded as important to find means to improve DC immunotherapy.

### Sialic acid in dendritic cell-T cell interactions

The ultimate function of DC immunobiology is the DC-T cell interaction, whereupon DCs present the uptaken, processed antigens to T cells, thus eliciting a specific, long-lasting immune response. Since immunological synapses between these two cells involve glycoprotein receptor-mediated process, it is, thusly, potentially influenced by sialic acid.

Dendritic cells’ sialic acid-containing glycans have been shown to negatively influence T cell priming, most likely by interference on MHC-mediated antigen presentation and co-stimulation (137, 138). In line with these findings, sialidase-treated moDCs were able to prime T cells and induce proliferation more efficiently than fully sialylated moDCs (3, 111). This effect could be attributable to the increased maturation by sialidase-treated moDCs (3). However, one should not discard a synergistic effect with enhanced protein–protein interaction due to the absence of the negatively charged sialic acid (137), leading to enhanced inter cellular interactions. The verified upregulation of a set of pro-inflammatory, Th1 profile-inducing cytokine expression (viz., IL-1α, -6, -12, and TNF-α) in sialidase-treated moDCs (with subsequent IFN-γ secretion) could also account for the observed increased priming.

Reinforcing these results, others have observed that endogenous sialidase activity also promotes cytokine production by moDCs and this has been attributable to the action of Neu3 upregulation during moDC differentiation (62). Interestingly, tolerogenic, immature moDCs present high sialic acid content, as well as regulatory T cells. Thus it has been hypothesized that, host-tolerance induction by DCs could be a Siglec-mediated process (93).

Taken together, this evidence reminds that DC sialylation has implications in the T cell interaction and it is likely to twist the immunogenic/tolerogenic balance. Thus sialylation should be considered to fine tune DC-based therapy either pathology-treating or tolerance-inducing.

### Dendritic cell glycan recognition of tumors

Dendritic cells functions also include specific identification of tumor cells and presentation of tumor antigens to T cells. One of the mechanisms for tumor cells recognition is through the binding of cells surface receptors to tumor-specific antigen (TSA), with an almost exclusive tumor expression and tumor-associated antigens (TAAs), normally expressed on the cells but of aberrant expression on tumor cells (139). Upon recognition, these antigens normally elicit a maturation response on DCs but the immune potency depends on many factors, including the antigen. Tumors have, however, several evasion strategies from immune responses, achieving this by creating a tolerance-inducing microenvironment, secretion of inhibitory factors, and activation of immunosuppressant intracellular pathways in the immune cells (140–142). DCs present certain flaws in their antigen-presenting strategy that tumor cells take advantage of in order to create defective T cell responses, thus creating problems in generating effective anti-tumoral solutions (142, 143).

Aberrant glycosylation is a hallmark of cancer cells and aberrant glycosylated proteins can be shed into the body fluids of the patients (serum, urine, pleural effusions, etc.). This altered glycosylation pattern in tumor cells includes either a loss or a gain of expression of certain glycan structures, the appearance of truncated structures, as well as of novel structures. Upregulation and/or downregulation of specific glycosyltransferases is often responsible for these changes. Tumor-associated carbohydrate (TAC) structures allow tumor cells to invade and metastasize or to evade the immune system. Immature and/or tolerogenic DCs can migrate to the rapidly growing tumor microenvironment, thus eliciting immune tolerance in several ways, such as T cell deletion, anergy, and T_reg_ activation (142–145). The tolerogenic profile depends on the DCs recognition and binding to TAC. However, how DCs recognize the tumor cells and in particular the TAC are not fully disclosed. The few available studies point, so far, to the CLRs, MGL-1, and DC-SIGN receptors as being relevant in tumor recognition and undesired tolerance induction (37, 146): the former is highly expressed in immature, tolerogenic DCs, and shown to interact with the tumor-associated Tn antigen-bearing forms of MUC1 (147); the latter is also expressed by immature DCs and recognizes Le^x^ and Lewis Y (Le^y^) glycoantigens in a carcinoembryonic antigen-context expressed in colorectal carcinoma. Besides these receptors, the observed involvement of DCs’ Siglecs (such as Siglec-3 and -9) could help justifying the frequent tolerance-induction mechanisms: by recognizing overexpressed sialylated antigens at the tumor microenvironment (e.g., sialyl T and sLe^a^ expressed on mucins), these receptors could send inhibitory intracellular signals from their ITIM motifs thus preventing DCs from differentiating (by inducing apoptosis of their precursors) or maturating, keeping them in a tolerance-inducing state with concomitant upregulated anti-inflammatory cytokine expression, downregulated pro-inflammatory cytokine expression, and reduced antigen-presenting capability (148–150).

It is now evident that TAC and in particular, sialic acid expression influences tumor progression. DCs become tolerogenic after recognition of TAC (including glycan-bearing/glycosylated TAC), favoring tumor progression and being generally associated with bad prognosis. The collected evidence regarding the glycan influence on anti-carcinogenic immune processes should be, therefore, seriously considered whenever DC-based immunotherapies against specific malignancies are available.

## Concluding Remarks

The weight of glycosylation and, in particular, sialic acid in biological processes is being increasingly acknowledged. Being at the terminal position of many glycans, it plays an essential role in modulating many of the DC functions. In human DCs, the majority of studies to date have focused on moDCs and only scattered and very scarce data was reported regarding other subsets. It would be extremely important to study these and other immune mechanisms from the newly identified subsets’ perspective and to complement those studies using mouse DCs other than the traditionally (myeloid) BMDCs. Being known that different subsets of DCs have different functions/affinities toward different pathogens/tissues (and elicit different responses) it should not come as a surprise that different subsets could express different glycans and glycan-recognizing receptors, having thus different underlying mechanisms and eliciting different immune responses. The discovery and accessibility of new, faster, and more precise glycobiology-related techniques may allow a better understanding of the role of sialylation and glycosylation in DCs. The problem that poses glycobiologists, and immunologists in particular, is trying to add a new perspective and knowledge, in the same magnitude, to the amount of knowledge that proteomics and genetics have gathered the last 30 or 40 years, in a short amount of time. That premise is getting growingly important every time a relevant role for glyco-based phenomenon is identified. Our hope, with this review is that we contributed a little bit more to put the spotlight on Glycoimmunology and encourage further investigations on this subject.

## Conflict of Interest Statement

The authors declare that the research was conducted in the absence of any commercial or financial relationships that could be construed as a potential conflict of interest.
